# Modified posterior lumbar interbody fusion using a single cage with unilateral pedicle screws: a retrospective clinical study

**DOI:** 10.1186/s13018-015-0243-3

**Published:** 2015-06-30

**Authors:** Chen Bingqian, Xue Feng, Shen Xiaowen, Zhang Feng, Fang Xiaowen, Qian Yufeng, Dong Qirong

**Affiliations:** Department of Orthopaedics, The Second Affiliated Hospital of Soochow University, Suzhou, Jiangsu China; Department of Orthopaedics, Changshu NO.1 Peoples’ Hospital Affiliated with Soochow University, 1# Shuyuan Road, Changshu, Jiangsu 215500 China; Department of Orthopaedics, Affiliated Hospital of Nantong University, Nantong, Jiangsu China

**Keywords:** Lumbar PLIF, Single cage, Unilateral transpedicular screws

## Abstract

**Background:**

The traditional posterior lumbar interbody fusion (PLIF) technique usually involves implantation of two cages through a bilateral approach and bilateral laminectomy, which requires bilateral transpedicle screw fixation. The procedure itself has several negative impacts. Therefore, a modified PLIF procedure that includes insertion of a unilateral cage through the symptomatic side with supplementary unilateral pedicle screws has been conducted.

**Materials and methods:**

Thirty-one patients with unilateral radiculopathy who were diagnosed with spinal stenosis along with degenerative disc disease and a herniated intervertebral disc with lumbar instability underwent a unilateral PLIF using a single cage and unilateral pedicle screws. The postoperative clinical evaluation was based on the visual analogue scale (VAS) and the Oswestry Disability Index (ODI) for back pain and leg pain at multiple time points following the surgery. Radiological assessments were performed with lateral plain radiographs taken preoperation, immediately postoperation, 1, 2, 3 and 6 months postoperation and at the most recent follow-up.

**Results:**

The patients all underwent a single-level fusion, and the mean duration for the surgeries was 94 min. The mean haemorrhage volume was 250 ml, and no blood transfusion was required for any of the cases. Twelve months postoperatively, all patients had achieved an Excellent or Good outcome (Excellent in 28 patients and Good in 3). The mean pain score was 6.8 prior to surgery and decreased to 2.3 at the 3-month postoperative examination. No significant complications or neurological deterioration occurred. None of the 31 patients appeared to have any fusion failure. No broken screw, screw loosening, significant cage migration or subsidence was observed in any of the cases. A mean increase in the intervertebral disc height of 3.14 mm from the preoperative measurement to the most recent follow-up examination was determined to be statistically significant (*p* = 0.05).

**Conclusions:**

Conducting PLIF using the diagonal insertion of a single cage with supplemental unilateral transpedicular screw instrumentation enables sufficient decompression and solid interbody fusion to be achieved with minimal invasion of the posterior spinal elements. This technique is a more clinically secure, straightforward and cost-effective way to perform PLIF.

## Introduction

Posterior lumbar interbody fusion (PLIF) is widely performed for patients suffering from degenerative lumbar spine diseases, such as spinal stenosis, lumber spondylolisthesis and lumber disc herniation with instability. PLIF has its advantages for restoring disc height, disc stabilisation, nerve root decompression and reinforcement of the anterior spinal column [1]. However, the traditional PLIF technique always involves implantation of two cages through a bilateral approach and bilateral laminectomy and requires bilateral transpedicle screw fixation for the initial stability [2, 3]. A study has reported that bilateral interbody cages and pedicle screw fixation can increase the successful fusion rate [4]. However, when inserting two cages, destroying posterior elements of the spine, such as the bilateral lumbar facet joint and lamina, is necessary, which can lead to iatrogenic instability of the posterior elements and can cause postoperative back pain syndrome [5]. Bilateral pedicle screw fixation can cause unnecessary trauma to the lumbar musculoligamentous complex and can consequently increase infection rates and lumbar musculoligamentous complex injury, which can result in poor clinical outcomes [5]. Additionally, a low medical cost-effectiveness is expected.

This study has retrospectively analysed the clinical and radiographic outcomes of performing unilateral PLIFs by inserting a single cage filled with an autogenously morselised bone via the symptomatic side and performing unilateral pedicle screw fixation.

## Materials and methods

Between January 2009 and September 2013, 31 patients with unilateral radiculopathy who were diagnosed with spinal stenosis with degenerative disc disease and herniated intervertebral disc with lumbar instability underwent a unilateral PLIF using a single cage filled with a local morselised bone graft via the symptomatic side and performing unilateral pedicle screw fixation.

The mean age at the time of surgery was 60.1 years (range, 45 to 65 years). The study population was 15 males and 16 females. All of the patients were ethnic Chinese, and the inclusion diagnosis was limited to unilateral radiculopathy caused by foraminal stenosis or degenerative disc disease, such as upper lumbar disc herniation, recurrent disc herniation and single-level lumber segment disorders. The problem segments were as follows: 17 cases with L4/5 and 14 cases with L5/S1. Patients with conditions requiring bilateral nerve root decompression and cage insertion, such as bilateral radiculopathy and spondylolisthesis and those with spinal osteoporosis, were excluded from the study.

All patients were followed for more than 1 year. The mean follow-up period was 24 months (range, 12 to 54 months).

### Surgical technique

**Fig. 1:**
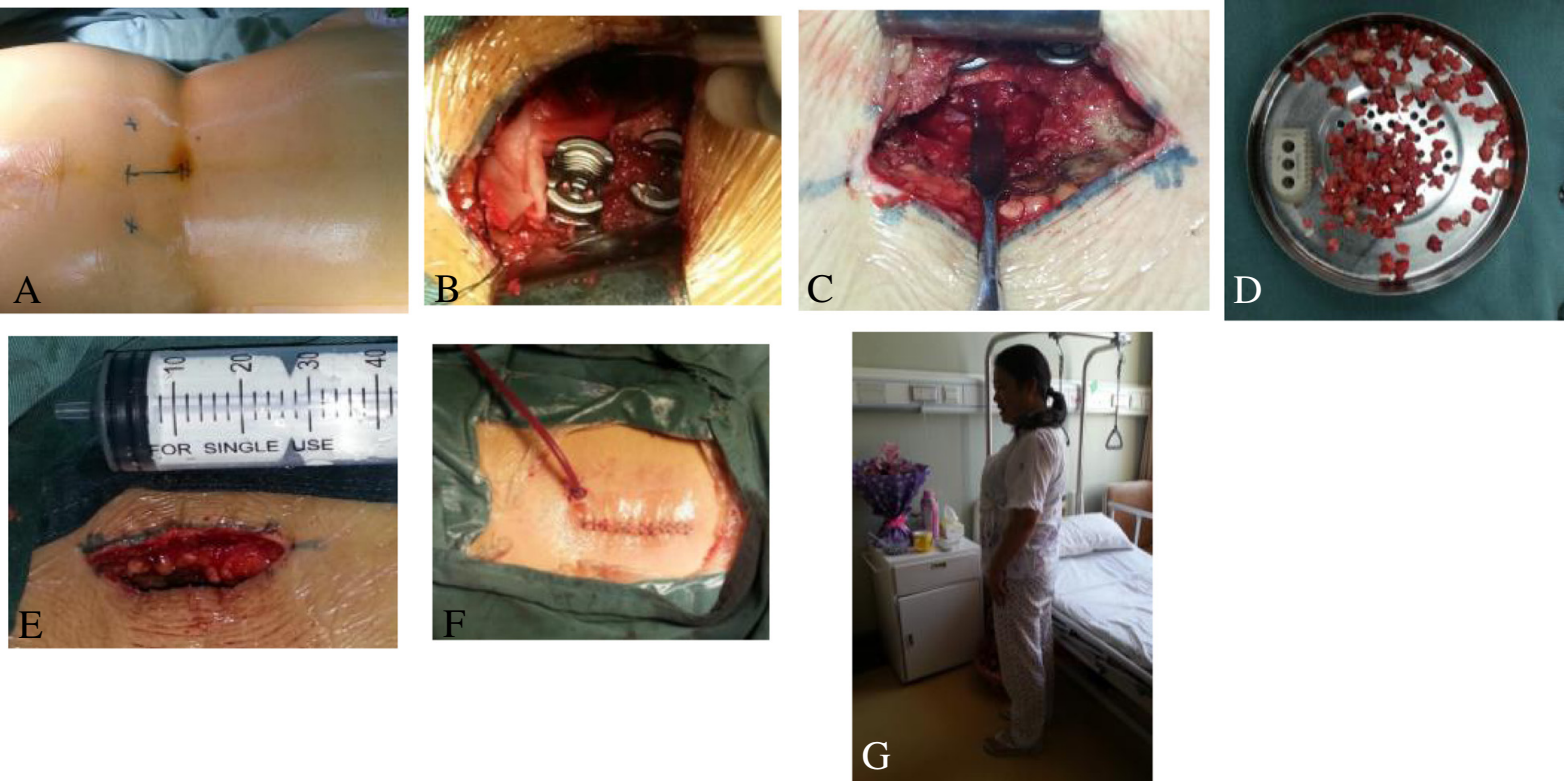
**a–g** Posterior lumbar interbody fusion using a unilateral cage with unilateral pedicle screws. **a** The patients were placed in the prone position under general anaesthesia. **b** The lamina and posterior joints of the symptomatic side were exposed, and a transpedicular screw system was placed under X-ray guidance. **c** Unilateral facetectomy and hemilaminectomy were performed on the symptomatic side, and the symptomatic nerve root was detected and decompressed carefully. **d** Autogenous morselised bone obtained from the laminectomy and facetectomy was inserted into the cage and disc space. **e**, **f** The length of the incision was not greater than 3 cm. **g** Three days after surgery, the patients could get out of bed and walk

**Fig. 2:**
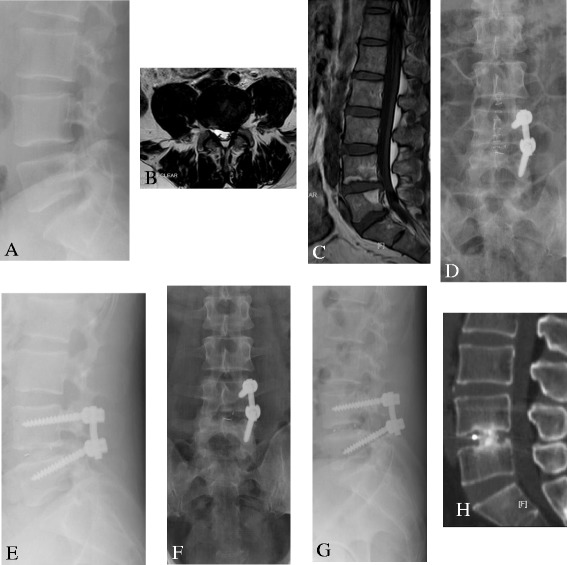
**a, b** A 46-year-old man suffered from lower back pain with radiation to the left leg. MR showed that the intervertebral disc for L4/L5 was herniated, and the left nerve root was compressed. **d, e**. The patient was treated with unilateral PLIF using a single cage supplemented with unilateral pedicle screws via the right side. **f**–**h** The radiograph and CT scan at 1-year follow-up showed bony trabeculae bridging the fusion level

All patients underwent unilateral single cage insertion and unilateral pedicle screw fixation. The patients were placed in the prone position under general anaesthesia. With the muscles adjacent to the spine on the symptomatic side retraced laterally to minimise damage, the area lateral to the lamina and the posterior joint was exposed. A transpedicular screw system was placed on the symptomatic side on the guide of the X-ray. Next, a unilateral facetectomy and hemilaminectomy were performed on the symptomatic side. The symptomatic nerve root was detected and decompressed carefully. Subsequently, the disc space for unilateral cage insertion was prepared with entire endplate curettage. The end plates of the central portion of the disc space were also curetted carefully. The contralateral disc space was filled as compactly as possible with autogenous morselised bone obtained from the laminectomy and facetectomy. Accordingly, a single cage filled with morselised bone graft material was inserted into the disc space. Eventually, the surgery was performed by compressing the intervertebral space slightly with pedicle screw fixation to secure stability and improve the bony union immediately postoperation. In the procedure, the spinous process, supraspinous and interspinous ligaments and the contralateral vertebral plate and facet joints, remained uninjured (Figs. 1 and 2)

Three days after the operations, the patients were able to get out of bed and walk with the protection of a functional waist brace, which was used for 4 weeks.

### Clinical outcome assessment

The postoperative clinical evaluation was based on the visual analogue scale (VAS) and the Oswestry Disability Index (ODI) for back pain and leg pain at multiple time points following surgery. The radiological assessment was performed with lateral plain radiographs taken preoperation, immediately postoperation, 1, 2, 3 and 6 months postoperation and at the latest follow-up.

The bony union was evaluated with careful assessment of the formation of bone bridging and the absence of radiolucency around the cages. A solid bony union was considered to be obtained when the endplates became invisible on the follow-up radiographs, and bony trabecular continuity and bone bridging were observed in the intervertebral space. Fusion failure was defined as the presence on anteroposterior and lateral radiographs of a definite radiolucent line around a cage or pedicle screw or more than 5° of motion on lateral flexion-extension radiographs. The height of the intervertebral disc space was calculated as the mean of the sum of the vertical distances between the anterior and posterior edges of the vertebral endplates.

The demographic data, clinical outcomes, fusion success and related complications were analysed. Intra- and postoperative blood loss, operation duration and postoperative hospitalised days were also recorded.

### Statistical analysis

Statistical analysis was performed using State 10.0. Mean values (MV) and standard deviations (SD) were calculated. Parameters were compared between time points using the Wilcoxon signed-rank test. A *p* value of 0.05 was considered to indicate statistical significance.

## Results

Twelve months postoperatively, all patients had achieved an Excellent or Good outcome (Excellent in 28 patients and Good in 3). The mean pain score was 6.8 prior to surgery and had decreased to 2.3 at the 3-month postoperative examination. The decrease remained 12 months postoperatively. No significant complication or neurological deterioration occurred during the 12-month follow-up.

The radiological outcomes were listed as follows. At the 12-month postoperative examination, none of the 31 patients appeared to have fusion failure, as defined by the presence of a definite radiolucent line around the cage or pedicle screws or greater than 5° of motion on dynamic flexion-extension views. CT scan showed bony trabeculae bridging the fusion level. No broken screws, screw loosening, significant cage migration or subsidence was observed in any of the cases. The intervertebral disc height significantly improved from 9.11 mm preoperatively to 13.1 mm immediately postoperatively and was 12.25 mm at the latest follow-up. The mean increase in the intervertebral disc height of 3.14 mm from the preoperative measurement to the latest follow-up examination was statistically significant (*p* <0.05) (Table 1).

**Table 1:** Pre- and postoperative data

	Preoperative	Postoperative
Mean pain score	6.8	2.3
Intervertebral disc height	9.1 mm	13.1 mm

The patients all had a single-level fusion, and the duration of the surgeries was 94 min (range, 80 to 120 min). The mean haemorrhage volume was 250 ml, and no blood transfusion was required for any of the cases. The mean hospitalisation period was 7 days (range, 5 to 10 days) (Table 2).

**Table 2:** Patient data

Males (*n*)	Females (*n*)	Segment L4/5 (*n*)	Segment L5/S1 (*n*)	Average blood loss (milliliter)	Average surgery time (minutes)	Average hospital stay (days)
15	16	17	14	250	94	7

## Discussion

PLIF not only relieves the pain resulting from nerve compression by neural decompression of the symptomatic side but also restores disc height, maintains vertebral alignment, restores weight bearing and reconstructs stability of the segment. PLIF has been reported to obtain a higher rate of fusion of the intervertebral segments and more satisfactory clinical outcomes than posterolateral bone grafting [6, 7].

The traditional PLIF technique is usually performed by inserting two cages via a bilateral approach with extensive laminectomy or posterior facetectomy and combining bilateral pedicle screws to provide spinal stability. The procedure itself has some disadvantages. Primarily, wide laminectomy, bilateral facetectomy and extensive intraoperative paraspinal muscle exposure around the posterior segments in the procedure increase the trauma and blood loss, causing denervation and atrophy of the paraspinal muscle, which results in a failed back syndrome [8]. Destruction of the bilateral facet joints and posterior ligament intraoperation can decrease spinal stability and hence increase the risk of perioperative or postoperative complications, such as cage migration [9]. In addition, the nerve root and dural sac always need to be retracted substantially to create two cages, which can cause bilateral nerve root injury or dural tearing. Eventually, additional bilateral pedicle screw fixation also requires contralateral extensive muscle release, which again increases trauma, blood loss and medical cost.

To solve the shortcomings of traditional PLIF, the technique has been modified in this study. Unilateral PLIF was performed by inserting a single cage filled with an autogenously morselised bone via the symptomatic side and unilateral pedicle screw fixation. The technique has several clear advantages over traditional two-cage PLIF supplemented with bilateral pedicle screws. First, one cage can be placed from the symptomatic side to avoid excessive retraction of the nerve root and dural sac of the asymptomatic side, which decreases the risks of epidural fibrosis and injury to neural structures related to retraction. Following the point mentioned above, in our technique, we only performed a partial laminectomy and facetectomy on the symptomatic side rather than a total laminectomy and bilateral facetectomy. The spinous process, supraspinous and interspinous ligaments, and the contralateral vertebral plate and facet joints are preserved in the procedure, which is very significant for maintaining stability of the lumbar spine [10, 11]. The destruction of the above structures always causes postoperative complications, such as cage migration and iatrogenic instability. Another important point is that the contralateral paraspinal muscle can be kept intact, decreasing blood loss, trauma and operative time. According to the report, excessive intraoperative paraspinal muscle exposure can also lead to denervation and atrophy, which results in a failed back syndrome. Finally, our procedure decreases medical cost by reducing the requirements for cages, transpedicle screws and transfusions.

The biomechanical tests on a calf lumber specimen after PLIF were conducted. The tests showed that the stability of the specimens with unilateral PLIF by inserting a single cage and unilateral pedicle screw fixation was weaker, but there were no significant differences than intact specimens. This finding suggests that this technology can provide adequate initial stability. Many studies have reported that one cage is enough in PLIF or TLIF. Oxland and Lund [12] also advised that single cage PLIF provides high stability in flexion; the supplementary use of pedicle screws improved stabilisation in all directions, and two-cage PLIF might increase the risk of damage to the bilateral nerve roots. Fogel [13] and Chang [14] reportedly suggested good results with unilateral cages and showed that patients with a unilateral cage had equal fusion and clinical success compared to those with bilateral cages. Zhao [15] noted that PLIF using a single threaded cage with a supplementary transpedicular screw and rod enables sufficient decompression and solid interbody fusion.

In our cases, satisfying clinical results and solid fusion union were both achieved. There were no complications, such as infection or neurological deterioration. No broken screw, screw loosening, significant cage migration or subsidence was observed in any of the cases.

All of our patients are ethnic Chinese who were lighter in weight and whose vertebral bodies were relatively smaller in size than those of Caucasians. The study does not provide sufficient evidence of whether this technique can provide adequate initial stability in Caucasian patients whose vertebrae are relatively bigger. During the interval, our patients only had unilateral radiculopathy caused by foraminal stenosis or degenerative disc disease. The patients with conditions requiring bilateral nerve root decompression and cage insertion, such as bilateral radiculopathy or spondylolisthesis and those with spinal osteoporosis, should choose traditional standard PLIF, and the unilateral PLIF techniques may not apply. Eventually, obese patients were not chosen for this surgery because of the expected risk that unilateral pedicle screws may not provide sufficient initial stability and hence lead to failure of the internal fixation.

## Conclusions

Unilateral PLIF with a single cage via a unilateral approach with supplementary unilateral transpedicular screws enables sufficient decompression and solid interbody fusion while maintaining intact posterior elements. This technique is ideal for the patients with severe symptomatic axial LBP with radiculopathy from disc herniation or stenosis. However, identifying applicable patients is crucial for the success of the surgery.
